# 

**Published:** 1883-08

**Authors:** 


NOTICE.

***All articles for publication, books for review, business communications, etc., must be sent to Editor, 2109 Chestnut Street, Philadelphia.***

